# No Evidence of Avian Influenza A H5N1 among Returning US Travelers

**DOI:** 10.3201/eid1302.061052

**Published:** 2007-02

**Authors:** Justin R. Ortiz, Teresa R. Wallis, Mark A. Katz, LaShondra S. Berman, Amanda Balish, Stephen E. Lindstrom, Vic Veguilla, Kathryn S. Teates, Jacqueline M. Katz, Alexander Klimov, Timothy M. Uyeki

**Affiliations:** * Centers for Disease Control and Prevention, Atlanta, Georgia, USA

**Keywords:** Influenza, human, influenza A virus, H5N1, influenza, human/diagnosis, tropical medicine, travel, dispatch

## Abstract

We reviewed reports to the Centers for Disease Control and Prevention of US travelers suspected of having avian influenza A H5N1 virus infection from February 2003 through May 2006. Among the 59 reported patients, no evidence of H5N1 virus infection was found; none had had direct contact with poultry, but 42% had evidence of human influenza A.

As of June 2006, the epizootic of highly pathogenic avian influenza A H5N1 virus among birds had spread to 3 continents (*1*). Sporadic human H5N1 cases characterized by severe respiratory disease with high case-fatality have been reported in 10 countries: Azerbaijan, Cambodia, Djibouti, Egypt, Indonesia, Iraq, People’s Republic of China, Thailand, Turkey, and Vietnam (*2*). Investigations have implicated direct contact with diseased poultry as the primary risk factor for H5N1 virus infection (*3**,**4*).

To date, H5N1 virus infections among poultry or wild birds in the United States have not been identified. However, US residents may be exposed if they travel to H5N1-affected countries. In February 2003, the Centers for Disease Control and Prevention (CDC) developed interim guidance for testing of suspected cases of H5N1 in returned travelers (*5*). CDC revised the recommendations in February 2004 (*6*). We report the results of investigations of patients with suspected H5N1 that were reported to CDC from February 2003 through May 2006.

## The Study

We retrospectively analyzed available data on US patients with suspected H5N1 that were reported to CDC by clinicians and public health departments from February 2003 through May 2006. Clinical and epidemiologic data about reported patients were communicated to CDC by telephone, email, and/or fax. For each patient, we assessed whether criteria for recommended H5N1 testing were met (suspected H5N1 case definition). The suspected H5N1 case definition had 2 components: the hospitalized case definition included severe respiratory illness and recent travel to an H5N1-affected country; and the ambulatory case definition included acute respiratory illness, contact with domestic poultry or a known or suspected H5N1 case-patient, and recent travel to an H5N1-affected country (*6*) (Figure). Contact was defined as proximity <1 m, and direct contact was defined as physical touching.

**Figure:**
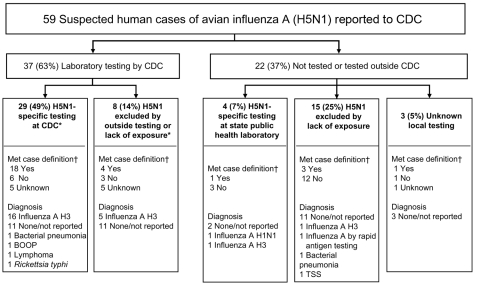
Influenza testing of suspected US cases of avian influenza A H5N1 reported to the Centers for Disease Control and Prevention (CDC) from February 2003 through May 2006. *Of the 37 samples tested by CDC, 35 were respiratory samples, 1 was serum, and 1 was a lung specimen. All 35 respiratory samples received by CDC were tested for human influenza by reverse transcription–PCR, and the serum sample was tested by microneutralization assay. †CDC suspected H5N1 case definition, February 2, 2004–June 7, 2006 (*6*): a patient is hospitalized and has radiographically confirmed pneumonia, acute respiratory distress syndrome, or other severe respiratory illness for which an alternate diagnosis has not been established; and the patient has a history of travel within 10 days of symptom onset to a country with documented H5N1 avian influenza in poultry and/or humans; or a patient is hospitalized or ambulatory and has a documented temperature >38°C (>100.4°F); and has a cough, sore throat, or shortness of breath; and has a history of contact with domestic poultry or a patient with known or suspected H5N1 case in an H5N1-affected country <10 days of symptom onset. BOOP, bronchiolitis and obliterans organizing pneumonia; TSS, toxic shock syndrome.

If a patient met the suspected H5N1 case definition, or if exposure data were incomplete and clinicians or public health authorities had persistent concerns, H5N1-specific testing was recommended by CDC. A standard case report form was completed by state health departments.

Diagnostic testing for patients with suspected H5N1 was performed at CDC, state laboratories, or both. Procedures for reverse transcription–PCR (RT-PCR) and microneutralization assay for H5N1 have been previously described (*7**,**8*). Epidemiologic and laboratory data were analyzed by using EpiInfo version 3.3.2 (CDC, Atlanta, GA, USA).

Fifty-nine patients from 26 states were reported to CDC for suspicion of H5N1 virus infection from February 2003 through May 2006 (Table and Figure). Nineteen (37%) were male (n = 52), and the median age was 47 years (n = 49, range 2–87 years). Of the 37 specimens from patients tested at CDC, none had evidence of H5N1 virus infection. CDC tested samples from 8 persons for human influenza A only, and 5 were positive. Among the samples from 22 (37%) patients not tested at CDC, 4 (7%) were tested for H5N1 at state public health laboratories, and all were negative. Of the remaining 18 (31%) patients, 15 were not tested for H5N1 and state testing data were unavailable for 3 (Figure).

On the basis of available information, 27 (46%) patients met the CDC suspected H5N1 case definition (Table). Fourteen (24%) had severe, acute respiratory illness with recent travel to an H5N1-affected country. Overall, 25 (42%) patients, including 2 of 4 who died, tested positive for human influenza A. In addition, 52% of the 27 patients who met the CDC suspected H5N1 case definition had samples that tested positive for human influenza A. Four influenza A cases occurred outside the US influenza season. Of the influenza A–positive patients, 10 had H3N2 viral isolates that were characterized at CDC. All isolates were similar to human influenza A virus strains concurrently circulating in North America.

**Table:** Characteristics of cases referred to CDC for assessment of H5N1 infection*

Case-patient characteristics (n = 59)	No. (%)
Met CDC suspect H5N1 case definition	27 (46)
Met hospitalized case definition criteria	14 (24)
Met ambulatory case definition criteria	13 (22)
Bird proximity <1 m	14 (24)
Direct bird contact	0
Contact with confirmed human H5N1 case	0
Onset outside US influenza season	7 (12)
Outcome	
Hospitalized	20 (34)
Deceased	4 (7)
Diagnosis†	
No	28 (47)
Negative influenza test result but ILI and influenza A (H3)–positive contact	8 (14)
Human influenza A (H3)	23 (39)
Human influenza A (H1N1)	1 (2)
Human Influenza A by rapid antigen test‡	1 (2)
Community-acquired pneumonia	2 (3)
Bronchiolitis obliterans and organizing pneumonia	1 (2)
Lymphoma	1 (2)
*Rickettsial typhus*	1 (2)
Toxic shock syndrome	1 (2)
Country visited§¶	
People’s Republic of China	21 (36)
Vietnam	18 (31)
Thailand	11 (19)
South Korea	5 (8)
Taiwan	3 (5)
Cambodia	2 (3)
Other#	6 (10)
No foreign travel	4 (7)
Unknown travel history	2 (3)

*CDC, Centers for Disease Control and Prevention; ILI, influenzalike illness. †Sum of percentages >100% due to rounding. ‡Clinical diagnosis of human influenza. The patient was reported to CDC for suspected H5N1 virus infection but was determined to lack risk factors to warrant H5N1-specific testing. §Not mutually exclusive. ¶As of June 15, 2006, highly pathogenic avian influenza A H5N1 has not been confirmed by the World Health Organization or World Organization for Animal Health (OIE) in Saipan, Saudi Arabia, Singapore, and Taiwan (*1*). Japan was declared free of H5N1 by OIE on July 12, 2004 (*1*), 13 mo before the visit by a reported patient. South Korea was declared free of H5N1 (*1*) at the time of a visit by a reported patient. #One each for Indonesia, Japan, Malaysia, Saipan, Singapore, and Saudi Arabia.

Other diagnoses included community-acquired pneumonia, bronchiolitis obliterans and organizing pneumonia, toxic shock syndrome, lymphoma, and rickettsial typhus (Table). Among 28 patients without a diagnosis, 8 (29%) tested negative for influenza but had influenzalike illness and contact with an influenza A (H3)–positive person.

Among all reported patients, 52 (88%) had traveled to >1 of 11 countries in Asia with either confirmed human H5N1 cases or H5N1 in avian species before illness onset (Table). Four (7%) patients with suspected H5N1 had not traveled outside the United States, but they had contact with recent travelers to Asia, and 1 had traveled to a country without confirmed H5N1 in poultry or wild birds. Although 14 (24%) reported having been <1 m of any live poultry or domesticated birds in Asia, none reported touching live poultry, domesticated birds, or recently butchered poultry. No patients with suspected H5N1 had contact with any confirmed or suspected human H5N1 case-patients.

## Conclusions

Our review of patients evaluated for H5N1 among returned US travelers through May 2006 indicates that the risk for H5N1 to US travelers has been extremely low to date. A high proportion of the reported patients had evidence of human influenza A virus infection, but none tested positive for H5N1. Although direct contact with infected poultry is the primary risk factor for H5N1 virus infection (*3**,**4*), H5N1 virus transmission has been low, even among persons directly exposed to poultry suspected of infection with currently circulating H5N1 virus strains (*9**,**10*). None of the patients reported to CDC had touched poultry, and 48% of persons with cases that met the CDC suspected H5N1 case definition had not been <1 m of birds during travel.

Our finding that 42% of patient with suspected H5N1 had human influenza A emphasizes the importance of considering this disease year-round in returned travelers with acute respiratory infections. Human influenza activity in tropical and subtropical countries occurs year-round outside the typical US influenza season (*11*) and is the most frequent vaccine-preventable infection among travelers from Europe to tropical and subtropical countries (*12*). Moreover, the effect of influenza disease in tropical countries is substantial; for example, rates of influenza-associated hospitalizations in subtropical Hong Kong approximate US estimates (*13*).

Annual influenza vaccination is the best way to prevent human influenza. Influenza vaccine effectiveness depends upon multiple factors, including the degree of similarity between the vaccine strains and those in circulation. Persons at high risk for complications from influenza who were not vaccinated during the preceding fall or winter should consider influenza vaccination, if available, before travel (*14*). However, no human H5N1 vaccine is currently available.

Our study is subject to several limitations. The reported patients may not be representative of all US travelers at risk for H5N1 among whom respiratory illness developed, but were persons for whom health departments and physicians sought CDC consultation. States may have evaluated travelers for H5N1 virus infection without notifying CDC. Additionally, some reported patients were not tested for H5N1 if available epidemiologic and clinical information suggested that H5N1 virus infection was unlikely. Clinical charts were not independently reviewed, and clinical, epidemiologic, and laboratory data were limited to that sent to CDC by state and local health departments. In many instances, the role of CDC was to exclude the diagnosis of H5N1, and further testing to establish a diagnosis other than influenza was not always performed.

Continued surveillance and testing for H5N1 is warranted, given the current H5N1 epizootic, the ongoing occurrence of human H5N1 cases globally, and the importance of identifying influenza A viruses with pandemic potential in the United States as early as possible. In 2006, CDC and WHO revised their definitions for suspected H5N1 cases (*15**,**16*). The revised CDC suspected H5N1 case definition now specifically requires the person to have touched poultry or to have had contact with a patient with a confirmed or suspected H5N1 case, and it more clearly defines an H5N1-affected country. The findings of our study support these changes. Guidance for the evaluation of patients with suspected H5N1 should continue to be evaluated as more epidemiologic data become available.
